# Association between the Advanced Glycosylation End Product-Specific Receptor Gene and Cardiovascular Death in Older Men

**DOI:** 10.1371/journal.pone.0134475

**Published:** 2015-07-30

**Authors:** Erik Biros, Corey S. Moran, Paul E. Norman, Graeme J. Hankey, Bu B. Yeap, Osvaldo P. Almeida, Leon Flicker, Richard White, Rhondda Jones, Jonathan Golledge

**Affiliations:** 1 Queensland Research Centre for Peripheral Vascular Disease, College of Medicine and Dentistry, James Cook University, Townsville, Australia; 2 School of Surgery, University of Western Australia, Perth, Australia; 3 School of Medicine and Pharmacology, University of Western Australia, Perth, Australia; 4 Department of Neurology, Sir Charles Gairdner Hospital, Perth, Australia; 5 Department of Endocrinology, Fremantle and Fiona Stanley Hospitals, Perth, Australia; 6 School of Psychiatry and Clinical Neurosciences, University of Western Australia, Perth, Australia; 7 WA Centre for Health and Ageing, Centre for Medical Research, Perth, Australia; 8 Department of Psychiatry, Royal Perth Hospital, Perth, Australia; 9 Department of Geriatric Medicine, Royal Perth Hospital, Perth, Australia; 10 Department of Neurology, The Townsville Hospital, Townsville, Australia; 11 Department of Vascular and Endovascular Surgery, The Townsville Hospital, Townsville, Australia; University of Miami, UNITED STATES

## Abstract

Advanced glycosylation end product-specific receptor (AGER) signaling has been implicated in atherosclerosis. The aim of this study was to evaluate whether a common genetic variation in the *AGER* gene is associated with cardiovascular (CV) death. We included 1304 older men who were genotyped for rs1035798:C>T, which is a single nucleotide polymorphism (SNP) mapped to the third intron of *AGER*. Cox proportional hazard analysis was used to estimate the association of rs1035798:C>T with CV death. In addition we analyzed total RNA extracted from carotid atherosclerosis biopsies of 18 patients that did or did not have recent symptoms of cerebral embolization by quantitative real-time reverse transcription PCR (qRT-PCR). The minor T-allele of rs1035798:C>T was found to be associated with CV death under dominant (HR = 1.43, 95% CI: 1.01–2.02, P = 0.04) and recessive (HR = 2.05, 95% CI: 1.11–3.81, P = 0.02) models of inheritance even after adjustment for traditional cardiovascular risk factors. No association was found between rs1035798:C>T and non-CV death. qRT-PCR results suggested that median relative expression of *AGER* isoform 1 and isoform 6 transcripts were approximately 6- (P = 0.01) and 2-fold (P = 0.02) greater, respectively, within carotid biopsies of symptomatic compared to asymptomatic patients. These data suggest that the minor (T) allele of rs1035798:C>T represents an independent susceptibility factor for CV death. The expression of *AGER* isoforms is different in atheroma from patients with recent symptoms. Further studies are needed to investigate if rs1035798:C>T influences the alternative splicing of *AGER*.

## Introduction

Cardiovascular disease (CVD) is the leading cause of mortality worldwide and thought to be responsible for approximately 30% of deaths [1]. Most cardiovascular (CV) deaths are attributed to coronary heart disease (CHD) and stroke [2]. Acute rupture of an unstable atherosclerotic plaque is thought to be an important cause of ischemic stroke and myocardial infarction [3]. The exact mechanisms triggering plaque rupture are not completely clear, however monocyte-derived macrophages are believed to play an important role [4–6]. Whereas classically activated M1 macrophages are thought to promote inflammation [7], alternatively activated M2 macrophages are believed to limit inflammation [8]. The M1 and M2 macrophages are characterized by abundant production of inducible nitric oxide synthase (iNOS) and arginase 1 (ARG1), respectively, which makes these relatively specific markers for macrophage polarization in atherosclerosis [9].

A growing body of evidence suggests that advanced glycosylation end product-specific receptor (AGER)-mediated cell signaling may be important in plaque stability [10–13]. AGER is known to interact with a broad spectrum of ligands and multiple signaling pathways, such as those activated by the high mobility group box 1 (HMGB1) protein (a non-canonical ligand of AGER), important in the establishment of chronic inflammation [14]. Consistent with this rs1035798:C>T, a non-coding single nucleotide polymorphism (SNP) in the *AGER* gene, has been associated with clinically significant manifestations of atherosclerosis, such as ischemic stroke [15]. Several splice variants encoding different isoforms, including full-length signaling and truncated soluble variants, have previously been described for *AGER* [16]. The soluble AGER isoforms are believed to be cytoprotective against excessive AGER signaling by acting as decoy receptors [17]. In this paper, we provide evidence for the association of the *AGER* SNP rs1035798:C>T with CV death and report differential expression of *AGER* isoforms within biopsies of carotid atherosclerosis.

## Materials and Methods

### Participants

In order to assess the association of rs1035798:C>T with CV death we examined a group of 1304 community-dwelling men aged ≥70 from the Health In Men Study (HIMS) who had been prospectively followed for a mean of ~5.5 years through linked data. The characteristics of HIMS participants have been described in details previously [18, 19]. The definitions of CVD risk factors such as hypertension, dyslipidemia, diabetes, CHD, and smoking were also previously described [20]. In brief, dyslipidemia, hypertension, and diabetes were defined by a history of diagnosis or treatment of dyslipidemia, hypertension, or diabetes mellitus, respectively. CHD was defined by a history of myocardial infarction, angina, or treatment for coronary artery disease. Smoking was defined by history of ever-smoking. Waist-to-hip ratio (WHR) was calculated from subjects’ waist and hip circumference that were measured in accordance with guidelines of the International Society for the Advancement of Kinanthropometry [21]. Participants were followed using the Western Australian Data Linkage System (WADLS) which provides electronic linkage to data from the death registry and hospital morbidity data system and has been shown to have excellent accuracy [22, 23]. Deaths due to cardiovascular diseases were identified from the death registry using ICD-10 codes in the range I00-I99 [24]. Carotid atheroma biopsies were collected in RNAlater solution (Ambion) from 18 patients undergoing carotid endarterectomy. Carotid artery atheromas were obtained from 11 subjects with recent symptoms of stroke or transient ischemic attack (TIA) and 7 asymptomatic patients. Total RNA was extracted using RNeasy Mini Kit (Qiagen) according to manufacturer’s instructions. Ethical approval was granted from the University of Western Australia and The Townsville Hospital and Health Services Committees and written informed consent was obtained from each participant.

### Genotyping

The human *AGER* is a highly polymorphic gene with more than 190 SNPs mapped to its locus on the 6p21.3 chromosome [25]. A number of SNPs in *AGER* have previously been tested for an association with a range of CVDs. However, most of these SNPs, including functional polymorphisms such as rs1800624, rs1800625, and rs2070600, have been reported to have no association with CVDs within a meta-analysis [26]. The rs1035798:C>T genetic variation, which is a less-studied SNP in *AGER* and located at the genomic sequence position 5878 (g.5878C>T; NG_029868.1), was selected for genotyping because of its relatively high level of heterozygosity in white populations, thereby allowing the detection of all genotypes in a relatively small number of individuals [25]. In addition, the SNP has been previously associated with CVD, although no functional significance of this intronic SNP has been established [15]. DNA of HIMS subjects and patients undergoing carotid endarterectomy was extracted from total blood samples collected in sodium citrate tubes using DNeasy Blood & Tissue Kit (Qiagen) according to manufacturer’s instructions. Genomic DNA was supplied to the Australian Genome Research Facility (AGRF Ltd, Australia) who performed genotyping of the HIMS subjects, using the Sequenom’s MassARRAY system that utilizes a homogenous MassExtend (hME–single base extension) reaction termed iPLEX GOLD. Genotype calls were made using SpectroTYPERTM RT software (Sequenom Inc., San Diego, CA, USA).

### Tissue expression

We used total RNA samples obtained from 18 patients with and without recent symptoms of cerebral embolization undergoing carotid endarterectomy. Four and 7 patients presented with ischemic stroke and TIA, respectively, while 7 patients were asymptomatic. Quantitative real-time reverse transcription PCR (qRT-PCR) was performed for two *AGER* isoforms, i.e. the full-length variant (isoform 1 or *AGER*) that represents the predominant *AGER* transcript and the truncated splice variant (isoform 6 or *esAGER*) that is the primary secreted isoform of AGER [27]. In addition, we assessed the expression of high-mobility group box 1 (*HMGB1*), a gene coding for a non-canonical AGER ligand (considered to be an important biological marker of inflammation [28]) and the arginase 1 (*ARG1*) gene, that is abundantly produced by M2 macrophages [29] (which are thought to play an important role in atherosclerotic plaque stability [5, 30]). The relative expression of selected genes in each sample was calculated by using the concentration-Ct-standard curve method and normalized using the average expression of the glyceraldehyde-3-phosphate dehydrogenase (*GAPDH*) gene for each sample using the Rotor-Gene Q operating software version 2.0.24 (Qiagen). *GAPDH* was chosen as the “housekeeping” gene since analyses showed its expression to be similar in carotid biopsies from symptomatic and asymptomatic patients. The QuantiTect SYBR Green one-step RT-PCR Kit (Qiagen) was used according to the manufacturer’s instructions with 20 ng of total RNA as template. All reactions were independently repeated in duplicate to assess the repeatability of the results and the mean of the two values for each sample was used for analyses. SYBR Green qPCR primers were designed using the AlleleID software (PREMIER Biosoft) for *AGER* isoform 1 (5’-GGTCATCTTGTGGCAAAG-3’ and 5’-CTCTTCCTCCTGGTTTTC-3’, reference sequence NM_001136), and *AGER* isoform 6 (5’-TCAGCATCAGCATCATCG-3’ and 5’-TTCTGCTTCCCTGACTTTATC-3’, reference sequence NM_001206940). QuantiTect Primer Assays (Qiagen) QT01002190, QT00068446, and QT00079247 were used for the *HMGB1*, *ARG1*, and *GAPDH* assessments, respectively. Mann–Whitney U test was performed to identify differences in expression levels of the selected transcripts between patients presenting with symptomatic and asymptomatic carotid artery disease. All computations were performed using the SPSS statistical package v.17.0.2. Statistical significance was defined at the conventional 5% level.

### *In silico* analysis of rs1035798:C>T

The rs1035798:C>T SNP is located in the third intron of the AGER gene (MIM 600214). Functional in silico analysis of this non-coding SNP was performed using the Function Analysis and Selection Tool for Single Nucleotide Polymorphism (FASTSNP) server and the Human Splicing Finder (HSF) v2.4.1 tool to identify the most likely functional effects of rs1035798:C>T on AGER [31, 32]. FASTSNP evaluates putative functional effects of SNPs, including changes to the transcriptional level and pre-mRNA splicing. A FASTSNP risk score of very high, moderate to high or low to moderate is assigned to the most likely functional effects. The HSF tool is designed to identify putative donor and acceptor splice sites, branch points (BPs), and cis-acting elements such as exonic splicing enhancer (ESE) and exonic splicing silencer (ESS).

### Statistical analysis of the genotype data

For the genotype analysis the outcome of interest was the occurrence of CV death. Cox regression analysis was used to model the association between rs1035798:C>T genotypes and multiple covariates with CV death under dominant, recessive, and additive models of inheritance. Our data set contained no missing values. Results are presented as hazard ratios (HR) and 95% confidence intervals (95% CI). Selection of covariates was based on clinical significance as described previously [33]. A dominant model measured differences between T-allele carriers and C/C homozygotes of rs1035798:C>T, while a recessive model compared T/T homozygotes with C-allele carriers of rs1035798:C>T. An additive model imposed a genetic structure in which each additional copy of the rs1035798:C>T minor (T) allele increased HR by the same amount. Hardy-Weinberg equilibrium was tested using HPlus v3.1 [34]. Cox analysis was performed using the SPSS Statistics 22. The date of birth and of last follow-up was used to define survival time and those participants that did not die were censored at the date of last data linkage. A similar analysis was performed to assess the association of rs1035798:C>T with non-CV death. Statistical significance was defined at the conventional 5% level. The available sample sizes had >80% power to detect the effect size (HR) of approximately 1.4 for the association of rs1035798:C>T genotypes with CV death. Power calculations were performed by using the PS: Power and Sample Size Calculation v3.0 software [35]. Finally, Kaplan-Meier curves were constructed to estimate the probability of CV and non-CV death in relation to rs1035798:C>T genotypes and compared using the Mantel-Cox log-rank test. For all analysis assessing this SNP as a risk factor for mortality the time from birth to death or data censorship was used.

## Results

### Participant characteristics

Genotyping was carried out in 1304 HIMS men. Baseline characteristics of HIMS subjects are shown in Table 1. Participants had a frequent history of ever smoking (882/1304, 68%), hypertension (554/1304, 42%) and dyslipidemia (483/1304, 37%). Over a mean follow-up period of 5.51±1.57 years 484 deaths occurred, of which 131 (27%) were due to fatal cardiovascular events (Table 1).

**Table 1:** Characteristics of subjects included in this study.

Characteristic	HIMS group	Gene expression group		
		Symptomatic	Asymptomatic	P
Number	1304	11	7	-
Males	1304 (100%)	9 (82%)	5 (71%)	0.65
Age (years)	72.64±4.06	69.16±9.45	72.45±6.19	0.54
Follow-up (years)	5.51±1.57	-	-	-
Total deaths	484 (37%)	0 (0%)	0 (0%)	-
CV deaths	131 (10%)	-	-	-
Age at CV death	82.65±4.59	-	-	-
Waist-to-hip ratio	0.96±0.06	1.00±0.24	0.91±0.11	0.64
Diabetes	106 (8%)	2 (18%)	2 (29%)	0.65
Hypertension	554 (42%)	8 (73%)	6 (86%)	0.59
Past stroke	80 (6%)	4 (36%)	0 (0%)	-
Transient ischaemic attack	N/A	7 (64%)	0 (0%)	-
Coronary heart disease	325 (25%)	3 (27%)	4 (57%)	0.26
Dyslipidemia	483 (37%)	7 (64%)	5 (71%)	0.77
Ever smoking	882 (68%)	11 (100%)	5 (71%)	0.14

Age, calendar age at entry-to-study; HIMS, Health In Men Study; P, two-sided P value; N/A, not available; (-), not applicable. Nominal variables are presented as numbers, while continuous variables are presented as mean ± standard deviation (SD). Nominal and continuous variables were compared between subjects in the gene expression study using Fisher’s exact test and Mann-Whitney U test, respectively.

### Cox proportional hazard analysis

The frequency of the rs1035798:C>T minor (T) allele was 0.21 (Table 2). The genotype distribution of rs1035798:C>T passed testing for Hardy-Weinberg equilibrium in HIMS men (P = 0.12; Table 2) and was therefore assessed for association with CV death. Multivariate analysis showed that the rs1035798:C>T minor (T) allele was significantly associated with CV death under dominant (HR = 1.43, 95% CI: 1.01–2.02, P = 0.04) and recessive (HR = 2.05, 95% CI: 1.11–3.81, P = 0.02) models of inheritance after adjustment for traditional CVD risk factors (Table 2). Analysis was adjusted for CHD, diabetes, dyslipidemia, hypertension, ever smoking, and WHR. To assess if rs1035798:C>T was specifically associated with CV death as opposed to other causes of death, an additional analysis was performed to investigate the association of rs1035798:C>T with non-CV death. No association was detected (Table 2).

**Table 2:** Association of rs1035798:C>T with death in HIMS subjects.

Inheritance	Genotype	N (%)	MAF	HWE	CV death			Non-CV death		
					HR	95% CI	P	HR	95% CI	P
Additive	C/C	830 (63.6)	0.21	0.12	-	-	-	-	-	-
	T/C	409 (31.4)			1.32	0.91–1.90	0.15	1.01	0.80–1.27	0.94
	T/T	65 (5.0)			2.27	1.20–4.28	0.01	0.97	0.57–1.67	0.92
Dominant	C/C	830 (63.6)	0.21	0.12	-	-	-	-	-	-
	T/C	409 (31.4)			1.43	1.01–2.02	0.04	1.00	0.81–1.25	0.97
	T/T	65 (5.0)								
Recessive	C/C	830 (63.6)	0.21	0.12	-	-	-	-	-	-
	T/C	409 (31.4)								
	T/T	65 (5.0)			2.05	1.11–3.81	0.02	0.97	0.57–1.66	0.91

N, number of individuals; MAF, observed minor allele frequency; HWE, Hardy-Weinberg equilibrium chi-squared test P value; HR, hazard ratio; 95% CI, 95% confidence interval. Adjusted for CHD, diabetes, dyslipidemia, hypertension, smoking, and WHR.

### Kaplan Meier-Analysis

**Fig 1:**
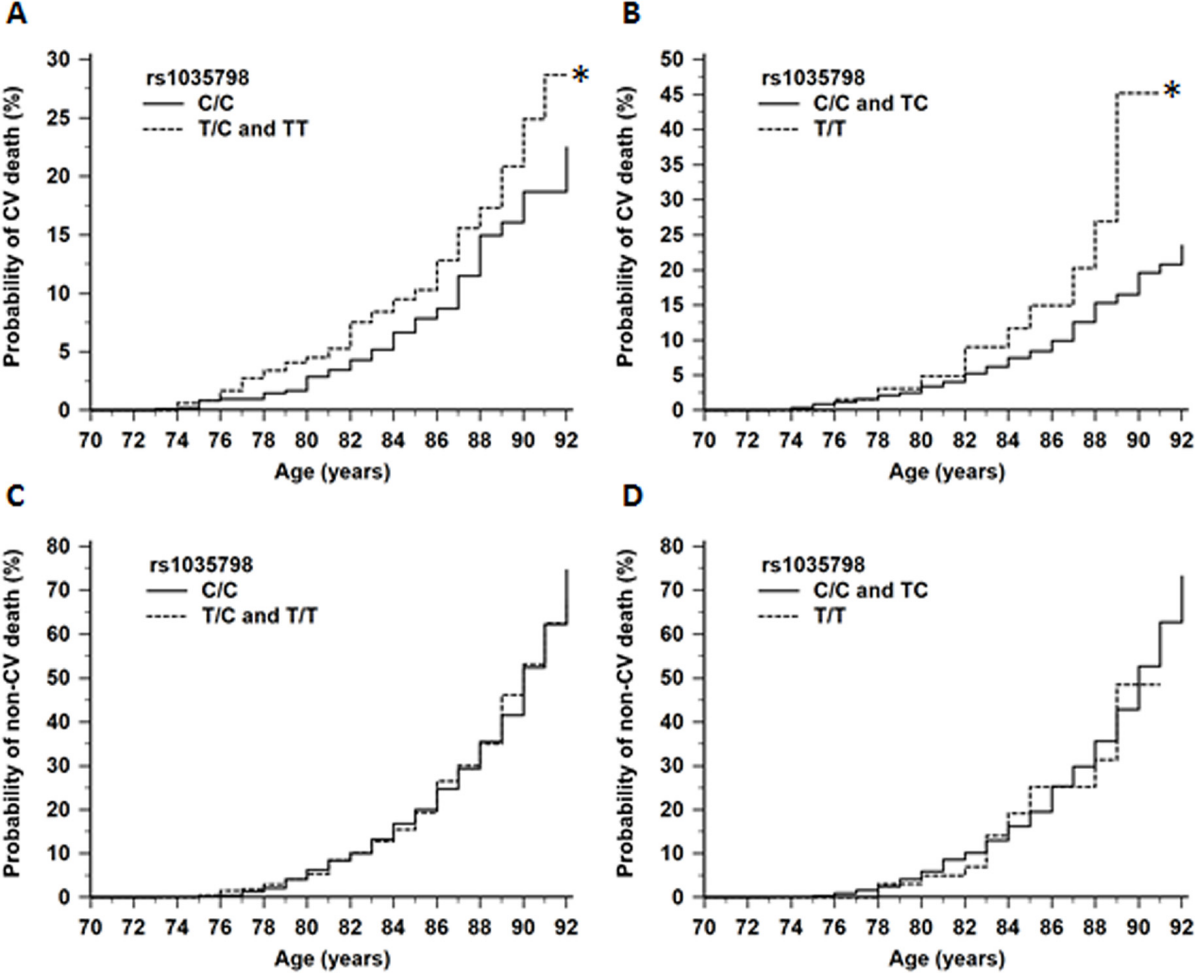
Kaplan-Meier analysis illustrating probability of death in relation to *AGER* rs1035798:C>T genotypes. The minor (T) allele of rs1035798:C>T was associated with an increased probability of CV death under a dominant (A; P = 0.04) and recessive (B; P = 0.03) model of inheritance. The T-allele had no association with the probability of non-CV death under a dominant (C; P = 0.95) and recessive (D; P = 0.87) model of inheritance.

The association of rs1035798:C>T with CV death was further examined using Kaplan-Meier analysis. The results are shown in Fig 1. An increased probability of CV death in relation to rs1035798:C>T minor (T) allele was seen under a dominant (P = 0.04; Fig 1A) and recessive (P = 0.03; Fig 1B) model of inheritance. The minor T-allele of rs1035798:C>T had no association with the probability of non-CV death (P>0.05; Fig 1C and 1D).

### Gene expression analysis

**Fig 2:**
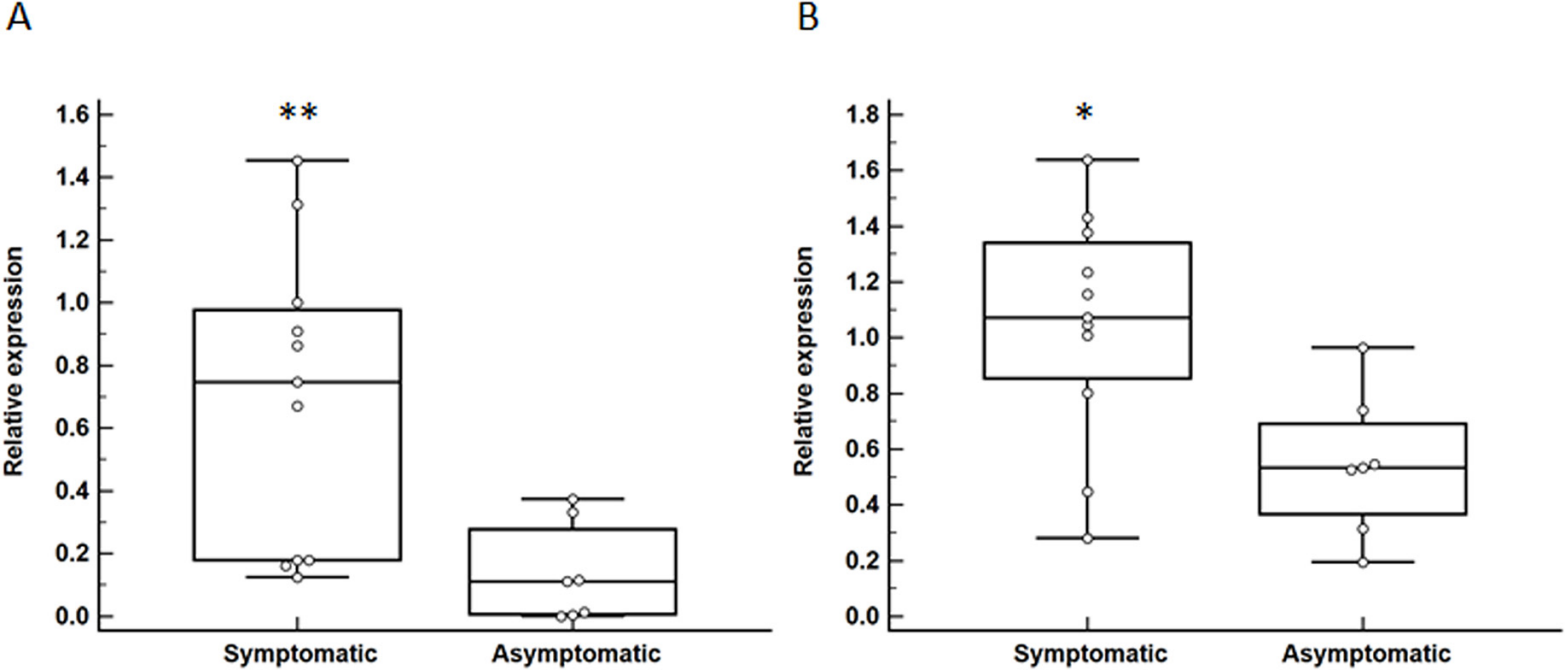
Differential expression of *AGER* isoforms in carotid atheroma biopsies from patients with and without symptoms of cerebral embolization. Expression of *AGER* isoform 1 (A; **P = 0.01) and *AGER* isoform 6 (B; *P = 0.02) was more than 6- and 2-fold greater within carotid atheroma biopsies of symptomatic compared to asymptomatic patients. Data expressed as median and interquartile range with maximum and minimum data points (whiskers) for relative expression.

**Fig 3:**
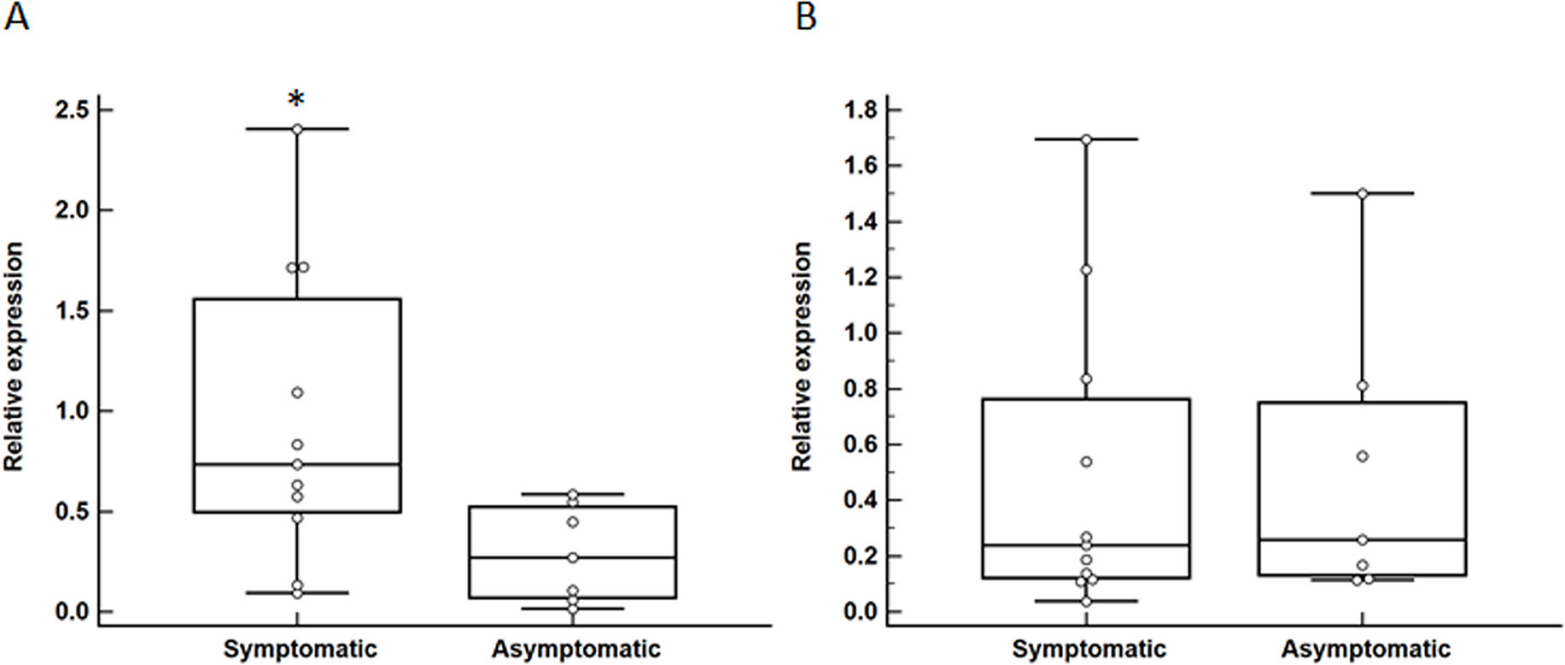
Differential expression of *HMGB1* and *ARG1* in carotid atheroma biopsies of patients with and without symptoms of cerebral embolization. Increased expression of *HMGB1* (A; *P = 0.02) but not *ARG1* (B; P = 0.82) within carotid atheroma biopsies of symptomatic compared to asymptomatic patients. Data expressed as median and interquartile range with maximum and minimum data points (whiskers) for relative expression.

A gene expression analysis was performed using carotid atheroma biopsies obtained from a group of 18 subjects comprised of 11 and 7 patients with and without recent symptoms of cerebral embolization, respectively (Table 1). Symptomatic patients presented with TIA (N = 7) and ischemic stroke (N = 4). Symptomatic and asymptomatic patients were similar in all of their baseline characteristics (Table 1). Median relative expression of *AGER* isoform 1 and isoform 6 transcripts were approximately 6- (P = 0.01) and 2-fold (P = 0.02) greater, respectively, within carotid biopsies of symptomatic compared to asymptomatic patients (Fig 2). Additionally, we assessed the expression of the *HMGB1* gene and found that median relative expression of *HMGB1* transcript was ~3-fold upregulated within carotid biopsies of symptomatic compared to asymptomatic patients (P = 0.02; Fig 3A). Finally, median relative expression of the *ARG1* transcript was similar within carotid biopsies of symptomatic and asymptomatic patients (P = 0.82; Fig 3B).

### *In silico* analysis of rs1035798:C>T

The *in silico* assessment of rs1035798:C>T was performed by using the FASTSNP and HSF bioinformatics tools. The rs1035798:C>T SNP was predicted to affect a splicing site with a moderate to high risk score by the FASTSNP software. Similarly, the HSF tool predicted that the rs1035798:C>T minor (T) allele breaks the recognition site for serine/arginine-rich splicing factor 5 (SRSF5) that is a member of the serine/arginine (SR)-rich family of pre-mRNA splicing factors.

## Discussion

We report an association between the *AGER* SNP rs1035798:C>T and CV death in older men. There was no association between this SNP and non-CV death. These findings are compatible with previous work on other inflammatory conditions such as multiple sclerosis (MS) assessed in white and African American MS cases and controls [36]. Although CVD and MS represent clinically distinct conditions, the association of rs1035798:C>T with these diseases would appear to be consistent across the different ethnic groups studied despite different allele frequencies. The frequency of rs1035798:C>T minor (T) allele is approximately 2% and 20% in African American and white populations, respectively [25]. AGER is a transmembrane signal transduction receptor with a number of ligands, including alarmins that can initiate and perpetuate immune responses [37]. AGER naturally exists in two forms that are full-length membrane-bound and truncated (soluble) [38]. The soluble form of AGER consists of several variants of different origin, including the endogenous secretory AGER (esAGER), which is a splice variant of the *AGER* gene transcript [38]. The esAGER protein contains all extracellular domains but not membrane and intracellular domains of the full-length AGER which preserves binding but not signaling capabilities [39]. We assessed the expression of *AGER* isoform 1 that codes for the full-length AGER protein and its splice variant *AGER* isoform 6, encoding the truncated esAGER protein, in samples of carotid atherosclerosis removed from patients undergoing carotid endarterectomy. Our findings that *AGER* isoform 1, and to a lesser extend *AGER* isoform 6 are upregulated within biopsies obtained from patients with symptoms compared to those without symptoms are in line with previous findings suggesting that AGER signaling is upregulated within vulnerable regions of atherosclerotic plaques [40]. The protein product of isoform 6 (esAGER) is believed to be protective against excessive AGER signaling [17], suggesting a role of this isoform in maintaining plaque stability. It has been proposed that alternative splicing of genes may correlate with sequence variations such as SNPs [41]. Our *in silico* analysis of rs1035798:C>T, an intronic SNP of *AGER*, predicted that the minor (T) allele of this SNP is expected to disrupt a recognition site for splicing factors that are involved in constitutive and alternative pre-mRNA splicing. It is possible that rs1035798:C>T influences the balance between *AGER* isoforms in atherosclerosis, however, this study does not identify a clear mechanism by which this SNP is associated with fatal CV events. The expression of *AGER* isoforms was not assessed in relation to rs1035798:C>T genotypes due to small number of carotid biopsies available.

Previous evidence suggests that complications of atherosclerosis result in part from the proteolytic activity of infiltrating inflammatory cells, notably macrophages, which promotes thinning of the fibrous cap and plaque rupture [5, 30]. In line with this, our current findings suggest marked upregulation of *HMGB1*, a gene coding for the non-canonical ligand of AGER, in patients with symptoms of carotid embolization. HMGB1 belongs to the group of endogenous damage-associated molecular pattern molecules (DAMPs), also known as alarmins. HMGB1 is passively released from necrotic cells and actively secreted from activated immunocompetent cells, including macrophages [42]. Extracellular HMGB1 has, however, inhibitory effects on phagocytic activity of macrophages (efferocytosis) that is critical to the resolution of inflammation [43, 44]. Consistent with this, Shaikh and co-workers showed by their comprehensive immunohistochemical assessments that unstable carotid plaques are characterized by a predominance of M1-like pro-inflammatory macrophages and a decreased proportion of atheroprotective M2-cells compared to femoral atheroma in humans [45]. Femoral atheroma has been suggested to be more typically associated with the stable flow-limiting symptoms rather than acute thromboembolism [46]. We found no evidence to support the association of M2 cells and carotid atheroma phenotype. We found similar expression of *ARG1*, a gene abundantly expressed by M2-macrophages [29], in patients that did and did not have symptoms of carotid embolization. In this context, previous evidence suggests marked upregulation of iNOS within unstable compared to stable human carotid plaques [47]. iNOS is abundantly produced by M1 macrophages, and importantly, this enzyme competes with ARG1 for the common substrate, L-arginine [48]. These and the current results collectively suggest an increase in the M1/M2 macrophage ratio in unstable atheroma and the possibility that L-arginine could have a plaque stabilizing effect. Possible pharmacological intervention may include cationic polyamines, such as poly-L-arginine, that can block the cellular uptake of L-arginine [49]. This, however, needs examining in other contexts, e.g. translational animal models that produce vulnerable plaques [50]. Currently there are no diagnostic or prognostic blood markers that can reliably identify patients developing ischemic complications of atherosclerosis. Our findings warrant further investigation with possible implications for the clinical management of atherosclerotic disease. Measuring circulating levels of AGER and HMGB1 may provide a useful clinical aid, the potential for which needs to be investigated.

The current study has a number of limitations. In order to assess the association of rs1035798:C>T with CV death, we included approximately 1300 participants prospectively followed over a mean period of ~5 years. Although CV death accounted for approximately 1 of every 4 deaths recorded in this study, the absolute number of 131 fatal CV events was relatively small, though sufficiently large to estimate statistically significant effect sizes. This, however, needs to be substantiated by larger studies and those involving different ethnicities. Detailed imaging of coronary, carotid, and cerebrovascular arteries was not performed. Additionally, all HIMS participants were men of largely white ethnicity limiting the relevance of our finding to women or other ethnicities. Recruitment of HIMS subjects focused on older men and thus our study is subject to healthy bias. It is therefore possible that the effect of rs1035798:C>T could be different if assessed in a cohort recruited at a younger age. Furthermore, we were only able to obtain proximal internal carotid artery biopsies taken from patients with and without recent symptoms of cerebral embolization for our gene expression study. The additional use of alternative samples such as coronary artery biopsies from patients undergoing coronary artery bypass grafting would have been useful to ensure the generalizability of our findings. Finally, the number of carotid artery plaque biopsies available for our gene expression study was limited which did not allow us to assess the differential expression of selected genes and isoforms in relation to rs1035798:C>T genotypes. Larger studies are required to assess this in detail.

### Conclusion

In conclusion, the current study suggests that rs1035798:C>T is associated with CV death. The finding needs validation in other cohorts.
